# Implant-type tissue-engineered cartilage derived from human auricular chondrocyte may maintain cartilaginous property even under osteoinductive condition

**DOI:** 10.1051/rmr/190001

**Published:** 2019-08-05

**Authors:** Natsumi Saka, Yoshinobu Watanabe, Satoshi Abe, Ayako Yajima, Hirotaka Kawano

**Affiliations:** 1 Department of Orthopaedics, Teikyo University School of Medicine, Tokyo, Japan, 2-11-1 Kaga Itabashi Tokyo 173-8606 Japan; 2 FUJISOFT Co., Ltd, Tokyo, Japan, 2-19-7, Kotobashi Sumida Tokyo 130-0022 Japan

**Keywords:** Cartilage, endochondral ossification, chondrocyte, cancellous bone, scaffold

## Abstract

*Introduction*: There is a growing need for chondrocyte implantation for reconstructing cartilage defect. However, ossification of the implanted cartilage is a challenging problem. Implant-type tissue-engineered cartilage from human auricular chondrocytes is a three-dimensional implant type cartilage using PLLA as a scaffold for chondrocytes. Although there is a study which evaluated the ossification of this cartilage in subcutaneous area, there is no study which clarify the possibility of ossification in osteoinductive surroundings. The purpose of this study was to elucidate the possibility of the ossification of implant-type tissue-engineered cartilage using human auricular chondrocyte in an osteoinductive environment. *Methods*: Human chondrocytes were harvested from ear cartilage. After dispersion by enzyme digestion, they were put into either a poly-L-lactic acid (PLLA) or poly lactic-co-glycolic acid (PLGA) scaffold, with collagen gel. Implant-type tissue-engineered cartilage was interposed between pieces of human iliac bone harvested from the same donor and implanted subcutaneously in nude rats. Scaffold without chondrocytes was used as a control. After 1, 3, and 6 months, ossification and cartilage formation were evaluated by X-ray, hematoxylin-eosin (HE) stain and toluidine blue (TB) stain. *Results*: There was no ossification of implant-type cartilage using human chondrocytes, even under osteoinductive conditions. HE staining showed that perichondrium formed around the constructs and chondrocytes were observed 6months after the implantation. TB staining showed metachromasia in every sample, with the area of metachromasia increasing over time, suggesting maturation of the cartilage. *Conclusions*: In conclusion, adjacent iliac bone had no apparent effect on the maturation of cartilage in implant-type tissue-engineered cartilage. Cartilage retention and maturation even in the presence of iliac bone could have been due to a scarcity of mesenchymal stem cells in the bone and surrounding area.

## 1 Introduction

In regenerative medicine, the reconstruction of cartilage is often the aim of treatment. Traditionally, for cartilage defects, autologous cartilage grafts from costal or knee cartilage have been widely used [15,13]. However, this procedure has major drawbacks, including donor site morbidity and a limitation on the amount of autograft available [6]. To overcome these problems for cartilage defects, the use of autologous chondrocyte implantation (ACI) has been increasing. ACI is mainly used for cartilage regeneration in the ear and nose, and articular surfaces. Several researches have already led to clinical applications [9,19].

One of the major problems with ACI, when it is used for cartilage regeneration, is endochondral ossification of the cartilage. Chondrocytes tend to undergo dedifferentiation, losing their ability to produce cartilaginous matrix and type2 collagen. After hypertrophic dedifferentiation, chondrocytes produce type1 collagen, and endochondral ossification, as seen in the growth plate, takes place. Some studies have reported ossification of ear cartilage autografts in rabbits [7,17]. Naumann reported mineralization of cartilage in vitro using human auricular and nasal chondrocytes [18]. Costales also reported ectopic bone formation in tissue-engineered cartilage derived from rabbit auricular cartilage [20]. This type of ossification has been the focus of recent studies in which bone has been regenerated from mesenchymal stem cell-derived chondrocytes or chondrocytes from nasal cartilage. There are a number of animal studies in which mesenchymal stem cell-derived chondrocytes were used for the reconstruction of large bone defects utilizing endochondral ossification [1,8,21]. Implant-type tissue-engineered cartilage was originally produced for the treatment of substantial nasal cartilage defects. When ACI was originally developed, a small volume of articular cartilage was harvested and digested with enzymes to obtain chondrocyte cells. Cultured cells in suspension were directly placed into the focal defect site and the site was covered by a periosteal patch to prevent leakage [2]. However, in more substantial cartilage defects with three-dimensional morphology, such as in ear and nose reconstruction, there is a need for cartilage with sufficient strength and shape.

Implant-type tissue-engineered cartilage was developed to overcome the lack of shape, strength and three-dimensional morphology in original ACI by Hoshi [5,22–24,27]. They used poly-L-lactic acid (PLLA) as a scaffold for chondrocytes. Harvested ear cartilage is digested using enzyme and chondrocytes are mixed with atelocollagen gel and then put into PLLA. PLLA biodegrades slowly, producing less foreign body reaction compared to other scaffolds and providing better surroundings for cartilage formation. By using PLLA as a scaffold, a three-dimensional, shaped cartilage with sufficient strength can be obtained. There is an ongoing clinical trial that is using this implant-type tissue-engineered cartilage for the reconstruction of nose deformity in cleft lip-nose patients [10].

Regarding the ossification of the implant-type tissue-engineered cartilage produced by Hoshi et al., there are animal studies which proved no bone formation or mineralization when the tissue-engineered cartilage was implanted in the subcutaneous tissue of nude mice, nude rats, and wild-type mice [5,14,24].

However, there are no studies which have evaluated ossification or cartilage regeneration of implant-type tissue-engineered cartilage when it is attached to the bone and for the application of tissue-engineered cartilage in various area, such study is needed. Cancellous bone has both osteoinductive and osteoconductive capacities, and some studies have reported bone formation when cartilage or hypertrophic cartilage was attached to subchondral bone [1,8,17,20]. On this basis, we have assumed that by making close contact with cancellous bone, an osteoinductive environment for cartilage can be produced.

Most of the studies which have evaluated ossification of cartilage have been in animal models, so to evaluate the possibility of clinical use, we used human-derived implant-type cartilage.

The purpose of this study was to elucidate that there is no ossification or calcification of implant-type tissue-engineered human-derived cartilage when attached to cancellous bone from the same donor and implanted in nude mice.

## 2 Materials and methods

### 2.1 Chondrocyte preparation

All procedures for the present experiments were approved by the Ethics Committee of FUJISOFT INCORPORATED (ethics permission #VII-2015·06-007). Implant-type tissue-engineered cartilage was made according to methods previously described. [9,10,23,27] Auricular cartilage and iliac bone, harvested from the same fresh cadaver, a 76 year-old male, were obtained through the Human & Animal Bridging Research Organization, Japan. The auricular cartilage was transferred to a container kept at between 1 °C and 10 °C, and the iliac bone was stored at −80 °C. After peeling away the perichondrium with a scalpel, auricular cartilage was cut into 1 cm^3^ pieces using scissors. The cartilage was then processed with 2.0–4.0 units of liberase (Roche, Basel, Switzerland) at 37 °C for 14 to 16 h. The isolated chondrocytes were seeded in a cell culture flask (Nippi, Incorporated, Tokyo, Japan) and cultured in Dulbecco's Modified Eagle's Medium Nutrient Mixture F-12 (DMEM/F-12) (Life Technologies Japan Ltd., Tokyo, Japan) containing 5% human serum(Sigma-Aldrich, St Louis, MO,USA), 100 unit/ml penicillin, 0.1 mg/ml streptomycin, 100 ng/mL fibroblast growth factor 2 (FGF2; Kaken Pharmaceutical Co., Ltd., Tokyo, Japan), and 5 mg/mL insulin in an incubator at 37 °C/5% CO_2_ for 13–14 days [12]. Passages were performed by treatment with trypsin-EDTA solution (Life Technologies Japan Ltd., Tokyo, Japan). Cultured chondrocytes were seeded again in a gelatin-coated fresco and cultured in the same medium in an incubator at 37 °C/5% CO_2_ for 19–21 days. After 19–21 days, chondrocytes were isolated with trypsin-EDTA solution.

### 2.2 Preparation and evaluation of iliac bone

The iliac bone, harvested from the same donor, was shaped into pieces the same size as the scaffold for implant-type tissue-engineered cartilage (5 × 5 × 3 mm^3^), and stored at −80 °C until it's use for implantation.

#### 2.2.1 Evaluation of cell survival

The iliac bone underwent freezing twice before implantation, at the time of harvest and at the time of shaping. In order to assess its viability after frozen storage, we separately evaluated cell survival at 47 and 63 days after harvest using iliac bones. At 47 days, the iliac bone was thawed and cut to yield 30 pieces 5 × 5 × 3 mm^3^. Fifteen pieces were evaluated immediately, in 3 groups of 5 pieces each. The other 15 pieces were re-frozen at −80 °C and evaluated at 63 days after harvest. To evaluate cell survival, five sections of iliac bone were irrigated with Hanks' Balanced Salt Solution with no calcium and no magnesium (HBSS(−); Life Technologies, Carlsbad, CA) at 37 °C for 10 min. Then they were processed with 1 mg/ml trypsin/HBBS(−) at 37 °C　for 10 min, centrifuged, and the cells then suspended in DMEM/F-12 with 10% FBS. Next, the cells were processed with 2 mg/ml dispase II (FUJIFILM WAKO Pure Chemical Corporation, Osaka, Japan)/HBBS(−) at 37 °C for 10 min, centrifuged, and suspended in DMEM/F-12 with 10% FBS. Finally, the cells were processed with 3 mg/ml Collagenase II (Funakoshi Co., Ltd.)/HBBS(−) at 37 °C　for 10 min, centrifuged, and suspended in DMEM/F-12 with 10% FBS. After staining with 0.5% trypan blue, the number of surviving and dead cells were counted using a disposable cell counter (One cell counter, OC-C-S02, One cell, Ltd). Cell survival rate was calculated based on the number of cells.

### 2.3 Fabrication of tissue-engineered cartilage and transplantation

Sections of PLLA and poly lactic-co-glycolic acid (PGLA) with dimensions 5 × 5 × 3 mm^3^ were used as scaffolds (GC Co., Tokyo, Japan). The molecular weight of the PLLA was 85,000–115,000, pore size was 0.15–0.8 mm, and average porosity was 87%. The molecular weight of the PLGA was 100,000–120,000, pore size was 0.15–0.8 mm, and average porosity was 80%. The human auricular chondrocytes at the third passage were suspended in 1% collagen hydrogel (atelocollagen, Koken, Tokyo, Japan) at a cell density of 5 × 10^7^ cells/ml. They were put into the scaffolds using a pipette and incubated at 37 °C/5% CO_2_ for 2 h, leading to gelation. The implant-type tissue-engineered cartilage was obtained by this procedure. This tissue-engineered cartilage was cultured in MEM (Life Technologies Japan Ltd., Tokyo, Japan), containing 5% human serum, 1 ng/mL FGF-2, 5 µg/mL insulin, 100 unit/ml penicillin, and 0.1 mg/ml streptomycin, incubated at 32 °C/5% CO_2_/60 rpm for 14 days. Once cultured, the tissue-engineered cartilage was placed between iliac bone pieces, using soft wire to hold the structure together.

Three types of construct were made for each of the two scaffolds (PLLA and PLGA): implant-type cartilage with iliac bone; implant-type cartilage without iliac bone; scaffolds (without cells) with iliac bone. Atelocollagen with FGF-2 and insulin was added to fill the void between the implant and iliac bone pieces. As controls, implant-type cartilage without iliac bone, and scaffolds without auricular cells placed between iliac bone pieces were used. For in vivo analysis, the constructs were subcutaneously transplanted into nude rats (F344/NJcl-rnu/rnu) (12-week-old, male) under general anesthesia (2% isoflurane, 0.4 L/min). Three skin incisions were made on the back and each type of construct was placed on the fascia. After 1, 3, and 6 months each construct was evaluated by X-ray. After X-ray, the rats were sacrificed and the samples were recovered. In total, six nude rats were used.

### 2.4 Radiological monitoring

Calcification was examined by radiological monitoring. Plain X-ray images were obtained under general anesthesia at 60 kV, two mA for 0.3 s using a Dexco ADX4000W (Osada medical Ltd. Japan) after implant transplantation and at the time of evaluation.

### 2.5 Histological evaluation

Each sample was fixed in 10% paraformaldehyde, decalcified with Plank–Rychlo's solution (0.5 M aluminum chloride containing 8.5% hydrochloric acid and 5.4% formic acid), embedded in paraffin, and cut into 5 µm sections. The sections were stained with hematoxylin and eosin (HE) to detect ossification as well as with toluidine blue to identify proteoglycan.

## 3 Results

### 3.1 Cell survival in iliac bone

At 47 days after harvest, the average number of living cells was 3.0 ± 0.82 × 10^4^ cell/ml, and the average cell survival rate was 93.3 ± 9.4%. At 63 days after harvest, the average number of living cells was 4.8 ± 2.9 ×1 0^4^ cell/ml, and the average cell survival rate was 91.0 ± 12.7%.

### 3.2 X-ray findings of the constructs

**Fig. 1:**
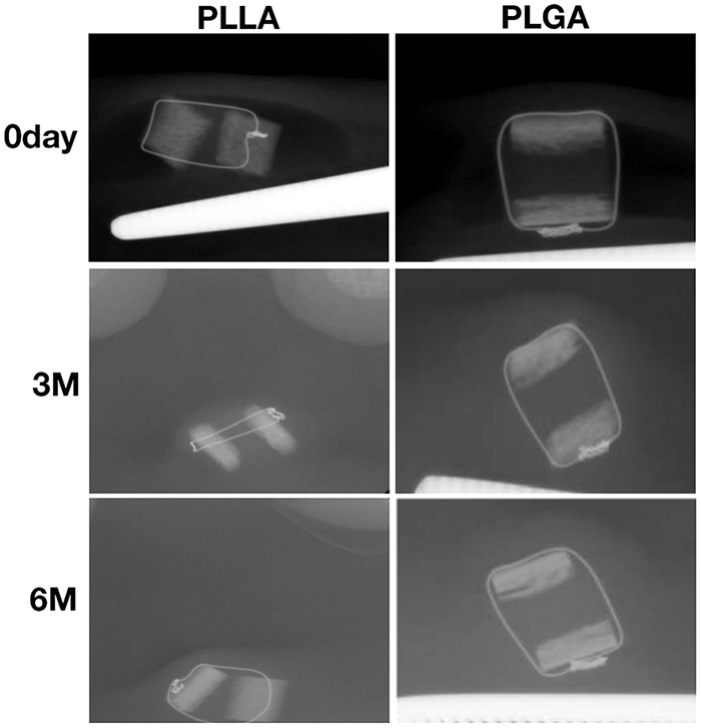
Findings of X-ray in PLLA/PLGA scaffolds with chondrocyte cells interposed with iliac bones. No calcification was observed in each sample.

On X-ray, no calcification was observed in any of the tissue-engineered cartilage interposed between the iliac bone pieces for either the PLLA or the PLGA scaffolds (Fig. 1).

### 3.3 Histological findings of the constructs

**Fig. 2:**
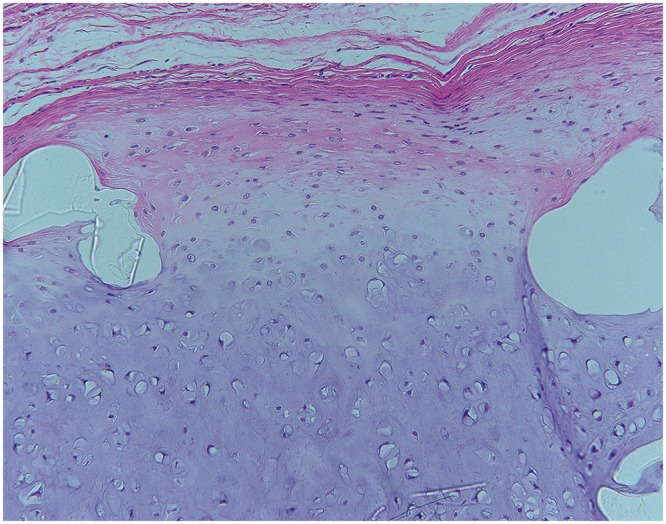
HE stain shows that perichondrium was formed around the constructs.

**Fig. 3:**
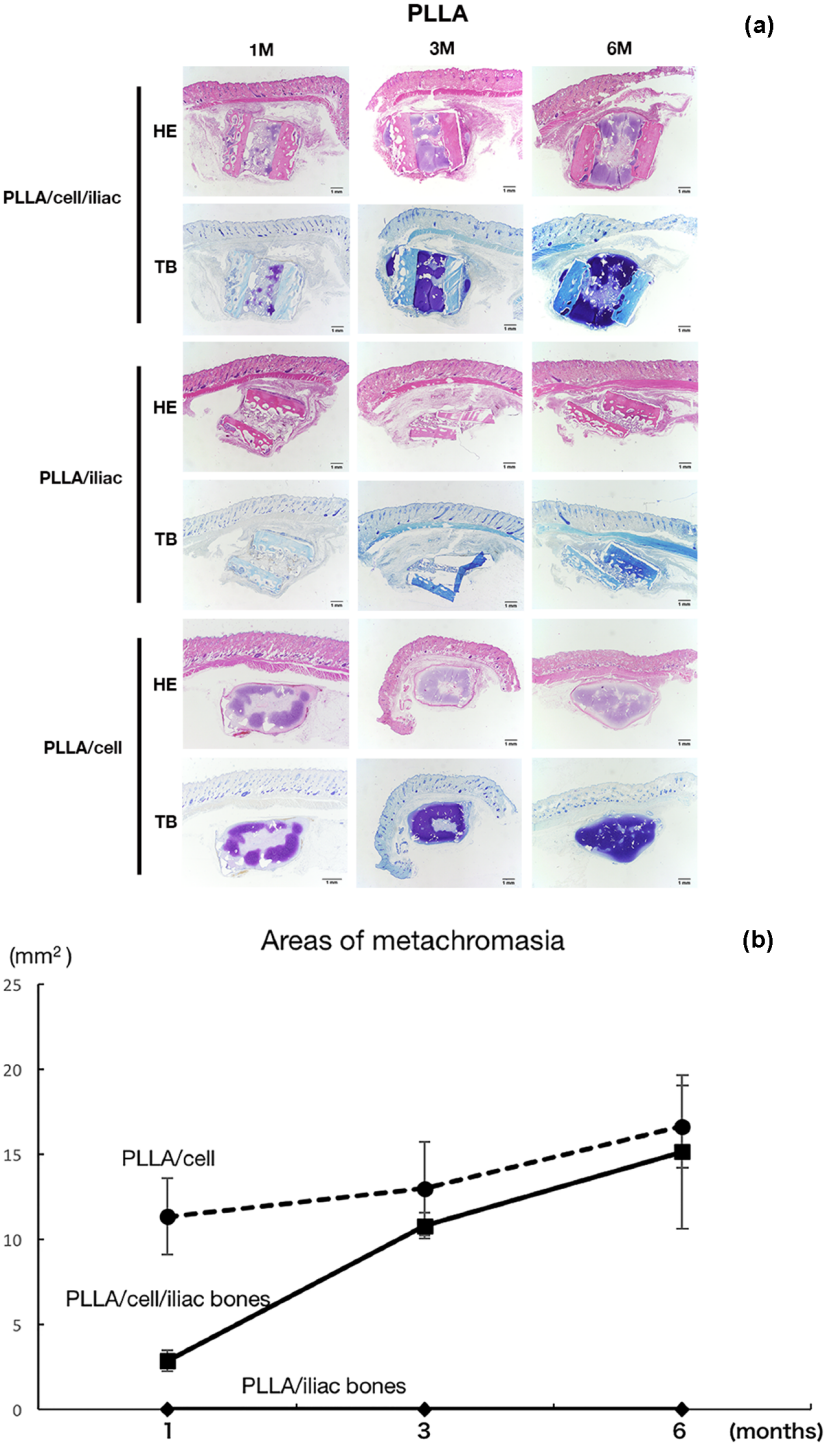
(a) Histological findings of Toluidine blue staining (TB) and Hematoxylin and eosin (H–E) staining (HE) in each group with PLLA scaffolds at 1, 3 and 6 months after the transplantation. HE stain showed that there is no osseous structures formation in each group. Proteoglycan was observed as metachromasia in the constructs with PLLA/cells/iliac and constructs with PLLA/cells in TB stain. (b) The observation revealed that the areas of metachromasia increase with time.

In the PLLA group, HE staining demonstrated that there was no ossification in the constructs in any sample throughout the experimental period. In the PLLA/cell/iliac and PLLA/cell groups, there was cartilage formation with perichondrium in the tissue-engineered cartilage (Figs. 2 and 3a). In the PLLA/iliac group, neither cartilage formation nor bone formation was confirmed and the PLLA scaffold had degraded over time (Fig. 3a). Toluidine blue (TB) staining showed metachromasia (indicating proteoglycan accumulation) in the PLLA/cell/iliac group and the PLLA/cell group from 1 month after implantation. At 6 months, metachromasia was observed throughout the implant, which is suggestive of maturation of regenerative cartilage (Fig. 3a). The area of metachromasia increased over the duration of the experiment (Fig. 3b).

**Fig. 4:**
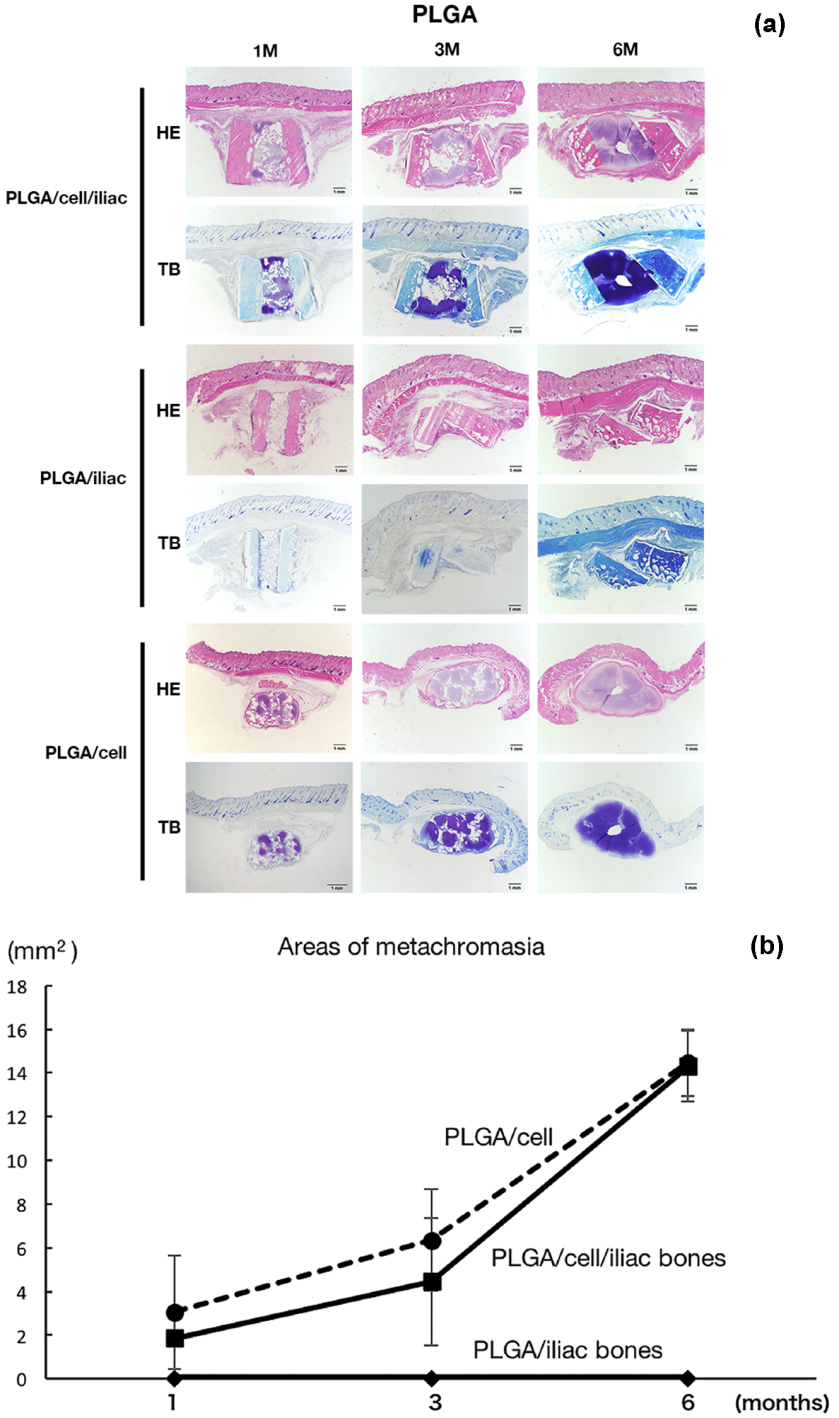
(a) Histological findings of Toluidine blue staining (TB) and Hematoxylin and eosin (H–E) staining (HE) in each group with PLGA scaffolds at 1, 3 and 6 months after the transplantation. HE stain shows that there is no osseous structures formation in each group. Proteoglycan was observed as metachromasia in the constructs with PLGA/cells/iliac and constructs with PLGA/cells in TB stain. (b) The observation reveals that the areas of metachromasia increase with time.

In the PLGA group, there was also no ossification throughout the period in any of the samples, as confirmed by HE staining (Fig. 4a). There was cartilage formation with perichondrium in the tissue-engineered cartilage in the PLGA/cell/iliac and PLGA/cell groups. Neither bone nor cartilage formed in the PLGA/iliac group. It was observed that the PLGA scaffold tended to degrade earlier than the PLLA scaffold. Toluidine blue staining showed metachromasia in the PLGA/cell/iliac and PLGA/cell groups 1 month after the implantation. At 6 months, metachromasia was observed throughout the implant (Fig. 4a). The area of metachromasia increased over the duration of the experiment (Fig. 4b).

**Fig. 5:**
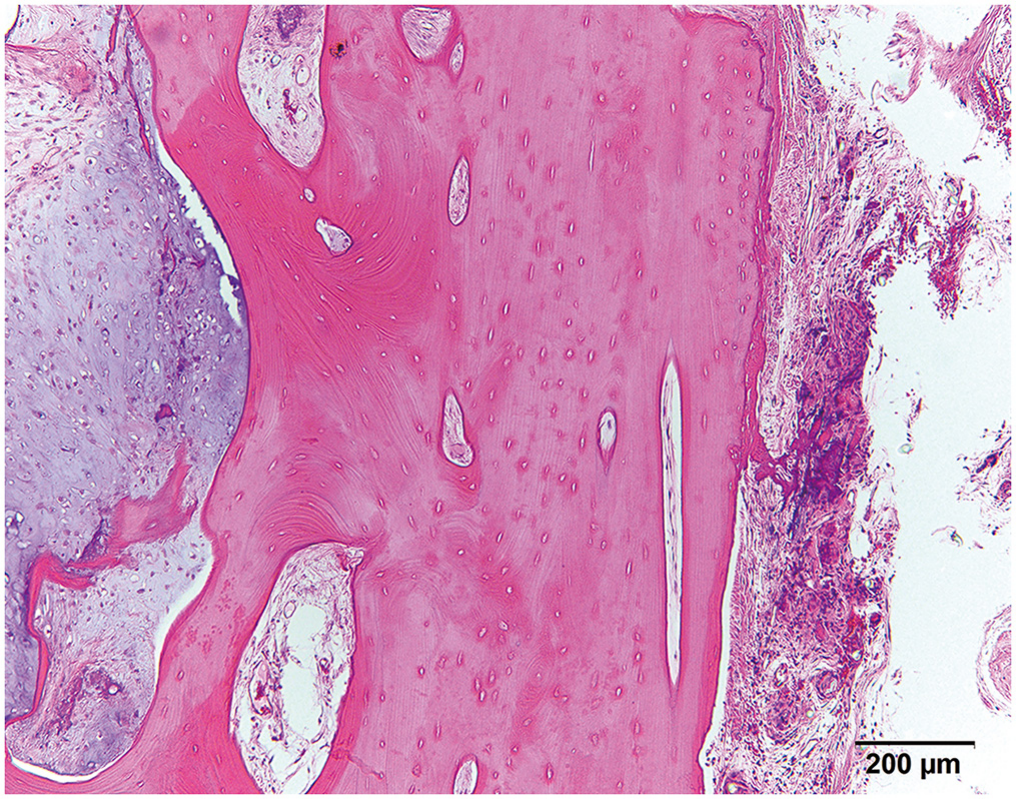
HE stain shows the remaining iliac bones at 6 months.

In all groups with iliac bones, the bone pieces retained their shape until six months after implantation (Fig. 5).

## 4 Discussion

This study showed that there was no bone formation or mineralization of implant-type tissue-engineered cartilage, and that mature cartilage was produced, even when it was interposed between iliac bone from the same donor.

In the process of endochondral ossification, at first, chondrocytes produce extracellular matrix, which acts as the initial cartilage template for ossification. As the chondrocytes undergo hypertrophy and apoptosis, osteoblasts differentiated from surrounding mesenchymal cells proceed to the zone of chondrocytes and start to form osseous tissue on the cartilage template. Mesenchymal stem cells are provided not only from bone marrow but also from other tissues, such as arterial and adipose tissue, and neovascularization occurs in the region concurrently [3]. In summary, for endochondral ossification, differentiation of chondrocytes, and recruitment of mesenchymal cells to the cartilage template followed by neovascularization are essential.

This process is controlled by several soluble factors. Bone morphogenetic protein-2 (BMP-2), which belongs to the transforming growth factor beta (TGF-β) family, has been proven to stimulate hypertrophic maturation of chondrocytes [25]. Inflammatory signals also play a role in triggering ossification. Interleukin 1 beta (IL-1β), produced mainly by macrophages, promotes angiogenesis and cartilaginous callus formation [16].

In the case of chondrocyte regeneration, some studies have shown successful cartilage regeneration, whereas other studies have revealed osseous tissue formation due to endochondral ossification or mineralization in the process of cartilage regeneration [7,17,18,20]. Bardsley reported osseous tissue formation using engineered hypertrophic cartilage made from chondrocytes when implanted in cranial bone defects [1]. Using the same type of implant-type cartilage used in our study, Fujihara reported the formation of mature cartilage in implants in the subcutaneous area of nude mice. No osseous tissue formation was observed in this study [5]. Costales used implant-type cartilage made from chondrocytes from rabbit ear cartilage, transplanting them either subcutaneously or into cartilage defects in the rabbit. There was no bone formation in the cartilage transplanted to the subcutaneous area whereas some parts of the cartilage transplanted into the cartilage defects showed the ossification [20].

Johnson reported up to 40% calcification of pig-derived chondrocytes implanted in nude mice and concluded that remnant subchondral bone played some role in the calcification. However, they did not discuss further the mechanism of calcification, which occurred in the cartilage adjacent to the bone [12]. Based on these findings, it is considered that environmental factors, such as the existence of mesenchymal stem cells originating from surrounding tissue, and soluble factors, such as IL-1β, played a role in the ossification observed.

Considering the role of mesenchymal stem cells, in our case, implant-type tissue-engineered cartilage was interposed between cancellous bone pieces implanted subcutaneously in rats an area that could be expected to act as a source of mesenchymal stem cells. Free iliac bone has often been used as bone graft material in the expectation of osteoinduction in a clinical setting, and the number of osteogenic progenitor cells in iliac bone is higher than other sites in the body, such as distal tibia or calcaneus [11]. We confirmed that approximately 95% of the bone cells survived, even after having been frozen. However, although we did not test for the existence of mesenchymal stem cells, our samples lacked periosteum, which contains stem cells with a high regenerative ability compared to cancellous iliac bone [4]. Consequently, it is possible that the concentration of osteogenic mesenchymal stem cells presence might have been insufficient for ossification.

Another consideration is the scaffold. Cell-to-cell contacts have some role in chondrocyte differentiation and N-cadherin is related to the differentiation [26]. However, we used atelocollagen gel for the scaffold, which leads to the dispersion of chondrocyte cells in the gel. In previous study, it was shown that the expression of N-cadherin was lower in the scaffold with atelocolagen gel compared to the scaffold without the gel, meaning decrease in cell-to-cell contact may maintain chondrogenic property of cells [27].

Regarding inflammatory response and soluble factors, using the same implant-type cartilage from human chondrocyte implanted in nude mice, and using atelocollagen gel and PLLA as a scaffold, Fujihara reported that mature cartilage was produced after eight weeks and macrophage and neovascularization were excluded from the cartilage area by Macrophage migration inhibitory factor (MIF). In control cases of PLLA without atelocollagen gel, there was an increase in the level of IL-1β, as well as the area of neovascularization and the number of macrophages. It was considered that the atelocollagen gel acted as a physical barrier to macrophages [5]. This physical barrier effect might have occurred in our implant-type cartilage as well, leading to a low level of inflammation and soluble factors that would otherwise cause ossification.

There might be another physical barrier for implant-type cartilage against ossification. Through HE staining, perichondrium was observed around the implant-type cartilage. It has been suggested that perichondrium has a protective role for cartilage [7]. Perichondrium might have acted as protection from surrounding mesenchymal stem cells and neovascularization.

## 5 Conclusion

Implant-type tissue-engineered cartilage interposed between free iliac bone pieces and infused by atelocollagen gel did not show ossification. It was thought that lack of mesenchymal stem cells and a reduced inflammatory reaction around the cartilage led to maturation of the cartilage rather than ossification of the construct. This finding could be of benefit in the control of calcification of tissue-engineered cartilage.

## Conflict of interest

The institution of N.S., Y.W., S.A. and H.K. has outsourced the cell processing to FUJISOFT Co., Ltd, Tokyo, Japan for conducting this study. The institution of A.Y. has operated cell processing for conducting the study as an outsourcing from Teikyo University.
